# Prevalence of Violence Perpetrated by Healthcare Workers in Long-Term Care: A Systematic Review and Meta-Analysis

**DOI:** 10.3390/ijerph19042357

**Published:** 2022-02-18

**Authors:** Alessio Conti, Alessandro Scacchi, Marco Clari, Marco Scattaglia, Valerio Dimonte, Maria Michela Gianino

**Affiliations:** Department of Public Health and Paediatrics, University of Torino, Via Santena 5bis, 10126 Turin, Italy; alessio.conti@unito.it (A.C.); alessandro.scacchi@unito.it (A.S.); marco.scattaglia@unito.it (M.S.); valerio.dimonte@unito.it (V.D.); mariola.gianino@unito.it (M.M.G.)

**Keywords:** violence, healthcare workers, long-term care, nursing home, home care, systematic review, meta-analysis

## Abstract

This systematic review and meta-analysis aimed to determine the prevalence of violence perpetrated by healthcare workers (HCWs) against patients in long-term care (LTC). For this purpose, five relevant databases were searched. Two reviewers extracted data from the included articles independently and assessed their quality. Overall and subgroup random-effects pooled prevalence meta-analyses were performed. A series of meta-analyses stratified by study quality were also performed due to high heterogeneity. Nineteen articles were included, physical restraint (22%; CI: 15–29), verbal abuse (22%; CI: 16–28), and neglect (20%; CI: 15–26) attained the highest overall prevalence, while sexual abuse was less reported (2%; CI: 1–3). The prevalence of witnessed violence is generally higher than those reported by HCWs, and patients and their relatives reported fewer cases of violence than HCWs. Differences in violence perpetrated among LTC settings were found. Neglect (64%; CI: 56–72) and financial abuse (7%; CI: 3–12) reported by HCWs were higher in home care, while verbal abuse (21%; CI: 7–39) reported by patients or their families was higher in nursing homes. Our findings highlight that violence perpetrated by HCWs toward patients represents a significant concern in LTC, suggesting the adoption of reliable monitoring approaches and provision of assistance to victims in reporting abuse.

## 1. Introduction

Recent demographic and epidemiological transitions in Organisation for Economic Co-operation and Development (OECD) countries have resulted in a significant increase in the need for long-term care (LTC). Indeed, advances in health care in recent decades have led to an increase in the ageing population, resulting in an expansion of chronic and disabling diseases [1,2]. These health transitions have also been associated with changes in social patterns, including smaller families, different residential models, and increased participation of women in the labor force [3]. Together, all such factors contribute to the growing utilization of LTC services, with considerable increase in healthcare resources utilization and costs [3].

The term LTC refers to a variety of services that assist in meeting the medical and non-medical needs of people with chronic illnesses and disabilities, or older persons who are unable to care for themselves for long periods. Such care can be provided at home, in the community, in assisted living facilities or nursing homes, and is focused on individualized and coordinated services promoting independence, patients’ quality of life, and meeting patients’ needs over a period of time [4]. Due to social and clinical impairments, LTC recipients are particularly vulnerable and, whether they are children or adults or older persons, constitute a significant part of the population exposed to the risk of violence perpetrated by healthcare workers (HCWs) [5].

Recent literature on violence has focused on elder abuse in general, and studies have been primarily conducted on older persons living in the community. In particular, a 2016 scoping review examining 18 community-based articles reported that the global 1-year prevalence of aggregated elder abuse ranged from 2.2 to 36.2%, with a mean of 14.3% [6]. Moreover, a 2017 review including 52 articles from 28 countries estimated a 15.7% global prevalence of elder abuse over the past year [7]. Growing emphasis has been placed on assessing the prevalence of elder abuse in specific LTC settings, such as nursing homes. In fact, it was estimated by a 2019 review, including nine articles, that rates of elder abuse are of particular concern in LTC settings where patients are institutionalized, accounting for 64.2% of HCWs reporting some form of abuse in the last year [8]. The studies that have focused on violence experienced by people with disabilities are dated. In 2012, two systematic reviews reported prevalence estimates of violence against adults (aged ≥ 18 years) or children (aged < 18 years) with disabilities. These reviews estimated the prevalence of neglect, physical, sexual, and psychological abuse perpetrated in the community while not determining who perpetrated the violence, which could also have been performed by family members or peers [9,10].

As aforementioned, the literature has investigated violence with some sector-specific focuses, either considering only older persons in community or residential settings, or people with disabilities primarily in community settings, and without identifying the specific category of abusers. Despite increasing attention to violence toward LTC patients, to date, a comprehensive systematic review and meta-analysis on the prevalence of such abuse is still missing. An estimate of the magnitude of violence from HCWs in all LTC settings, with a distinction among HCW’s self-reported, witnessed, and abuse reported by patients or their family members, could guide healthcare policies toward designing preventive interventions intended to control this phenomenon.

Thus, this systematic review aimed to determine the prevalence of violence perpetrated by HCWs against patients in LTC, including both residential and home settings.

## 2. Materials and Methods

This systematic review and meta-analysis is reported in accordance with the Preferred Reporting Items for Systematic Reviews and Meta-Analyses (PRISMA) guidelines. The protocol for this systematic review has been preregistered on Prospero (https://www.crd.york.ac.uk/PROSPERO/display_record.php?RecordID=162855 (accessed on 16 February 2022); registration number: CRD42020162855). As this study presents aggregate data extracted from primary studies, thus preserving the same aim of the original studies, no ethical approval was applicable.

### 2.1. Inclusion and Exclusion Criteria

To be eligible for inclusion in this systematic review, studies had to be primary investigations conducted in a sample of adult patients using LTC services in an institutional (e.g., nursing home) or home care setting. In this systematic review, patients were intended as people in charge of LTC services without any restrictions concerning their underlying disease (e.g., dementia, physical disability), age, gender, or ethnicity. HCWs were intended as a broad term including both paid professional and paraprofessional HCWs. Among professional HCWs, physicians, registered nurses, and allied professionals (e.g., social workers, physiotherapists) were considered. Paraprofessional HCWs were intended as paid employees providing direct care to patients (e.g., nurse aides, nurse assistants). People providing direct care to patients but not paid (e.g., volunteers, informal caregivers) were excluded [11]. No restrictions were imposed with regard to the country in which the primary research was conducted.

All original articles that reported patients’ exposure to violence perpetrated by a healthcare worker (i.e., type I violence) in a LTC setting were included. Violence was defined as outlined by Phillips et al. [12], dividing it into seven domains: physical abuse (willful infliction of physical pain or injury), physical restraint (inappropriate or unauthorized use of physical limitation to control someone), verbal abuse (name-calling, yelling, swearing, intimidating, or threatening someone), psychological abuse (infliction of mental anguish including involuntary seclusion, denial of visitors, or lack of privacy regarding telephone or mail), sexual abuse (infliction of nonconsensual sexual contact of any kind, included sexual harassment and assault), neglect (failure to provide services or goods necessary to avoid physical harm, mental anguish or illness, including being ignored or treated with indifference), and financial exploitation (illegal or unethical misappropriation of funds, property, or assets of the individual).

This systematic review has considered only violence perpetrated in the LTC setting. The primary outcomes examined were the prevalence rate and characteristic of violence. Violence perpetrated by HCWs in hospital settings (i.e., general, specialized) were excluded. Violence toward HCWs perpetrated by patients, family members (Type II violence), or coworkers (Type III violence) were not considered. Articles were included if published in English or Italian, while those published in non-peer-reviewed journals as well as grey literature were excluded. No limit on the publication date was set.

Observational studies, including cross-sectional studies, case-control studies, and cohort studies, were included in this systematic review. Descriptive observational study designs were considered, and these included descriptive cross-sectional studies, individual case reports, and case series. Quantitative results of mixed-method studies were included if data could be extracted. Qualitative studies were excluded.

### 2.2. Search Strategy

A preliminary search was carried out on PubMed to identify the most appropriate terms to be used in the search. Subsequently, PubMed, Cumulative Index to Nursing and Allied Health Literature (CINAHL), APA PsychInfo, ISI Web of Science, and Embase Database were systematically searched from inception to March 31, 2021, to identify all the available literature on violence perpetrated by HCWs in LTC. The most important search terms used throughout the different databases were violence, abuse, mistreatment, healthcare workers. The following specific terms were used to explore the long-term care context: nursing home, home care, assisted living. Free and thesaurus terms were combined to incorporate all possible terms pertaining to the phenomenon. The search conducted on PubMed is reported as an example in Table S1—Supplementary Material. Electronic databases were searched to identify articles to be included. To this aim, reference lists of relevant articles were further scanned. An expert librarian collaborated in the search process.

Firstly, the records retrieved were checked for duplicates, and then the titles and abstracts of the citations identified were checked against the inclusion and exclusion criteria by two independent reviewers. The texts of previously identified articles were read in full and checked for final inclusion by two independent reviewers. Reasons for exclusion were noted transparently. Any disagreements arising at any stage of the review process were resolved through discussion with a third reviewer.

### 2.3. Data Extraction and Quality Assessment

Two reviewers (AS, MS) extracted data independently. A third reviewer (AC) checked extracted data to reach 100% agreement. The data extracted covered author(s), year of publication, the country in which the study was performed, the title of the article, study design, study sample, setting (subdivided in nursing home or home care), type of violence (categorized into the previously presented domains) [12], data source (subdivided in HCWs self-reported, HCW witnessed, and patient and family self-reported), and prevalence rates of violence. For the purposes of this study, the data source consisted of structured tools in the form of questionnaires, data reporting tools, surveys, and administrative databases. The frequency of different types of violence perpetrated either directly or witnessed was self-reported by HCWs, patients, and their relatives.

Articles in which violence was not reported according to the type of violence, categorized as physical abuse, physical restraint, verbal, psychological, sexual, neglect, and financial exploitation, were grouped by reviewers (AS, MS) under those categories. In the same way, if articles did not report a cumulative prevalence for each violence category, the prevalence rate was adjusted for the maximum number of possible responses for a category. For example, if multiple items in an article were examining verbal violence (e.g., yelling, intimidating, and name-calling), the number of cases reported in those items were summed up to obtain a numerator (total episodes of violence reported). Subsequently, a denominator was obtained by summing up the number of total responses that could be provided for each of these items. Two reviewers (AS, MS) conducted this process and were validated by a third reviewer (AC).

The quality appraisal of included articles was assessed using the checklist for studies reporting prevalence data developed by the Joanna Briggs Institute [13]. This nine-item questionnaire provides an evaluation of methodological quality examining the appropriateness of the (i) sample frame in addressing the target population, (ii) sampling process, (iii) sample size, (iv) description provided for the study subjects and setting, (v) data analysis coverage of the identified sample, (vi) methods used for the identification of the condition, (vii) reliability of measures applied, (viii) statistical analysis, and (ix) response rate management. This instrument is widely used during systematic evidence review, and focus on concepts that are key to an article’s internal validity; it could range from 0 to 9, with higher scores representing higher quality. For the purposes of this systematic review, articles that attained a score from 1 to 4 were considered of poor quality, those with a score from 5 to 7 were considered of fair quality, while we judged of high quality those articles with a score of 8 and 9. Two reviewers (AS, MS) assessed the quality independently, and any disagreement was resolved by discussion.

### 2.4. Statistical Analysis

#### 2.4.1. Overall Pooled Prevalence of Violence in LTC

The heterogeneity of prevalence estimates was assessed by computing the I^2^ index and performing the Cochran Q test before running the overall prevalence meta-analysis. An I^2^ > 50% and Cochran Q test significance *p*-values < 0.05 constitute a high degree of heterogeneity. High heterogeneity was expected; thus, a random-effects meta-analysis with 95% confidence intervals (CI) was performed for each of the seven violence categories. The meta-analyses were stratified by data source (HCWs self-reported, HCW witnessed, or patient and family self-reported) to identify any possible difference due to violence reporting. Because in highly heterogeneous meta-analyses, the random-effects model still results in a high mean square error, a series of meta-analyses stratified by study quality was also performed. This assessment provided more robustness and led to the correct interpretation of probability of confidence interval coverage, regardless of heterogeneity. As data were non-normally distributed, a Freeman–Tukey double arcsine transformation was used to synthesize the proportions collected from the included articles. This approach was selected to stabilize the variance of data [14].

#### 2.4.2. Subgroup Meta-Analysis of the Prevalence of Violence in Different LTC Settings

A series of subgroup meta-analyses was conducted to determine potential sources of heterogeneity. Such meta-analyses were performed to assess the prevalence of each type of violence between long-term settings, subdivided into nursing homes and home care. Each meta-analysis was stratified by data source (HCWs self-reported, HCW witnessed, or patient and family self-reported). Data from at least three studies have to be extracted to perform subgroup analyses.

A meta-regression was also performed to assess the association of the characteristics of each setting. Data on setting characteristics should be available from 10 and 20 articles respectively to perform a univariate or multivariable meta-regression. Sensitivity analyses were performed by repeating the main meta-analyses for each type of violence, excluding one study at a time. The presence publication bias was assessed through visual inspection of the funnel plots and performing the Egger test [15]. Analyses were conducted using Stata version 17 (StataCorp. 2021. College Station, TX, USA).

## 3. Results

**Figure 1:**
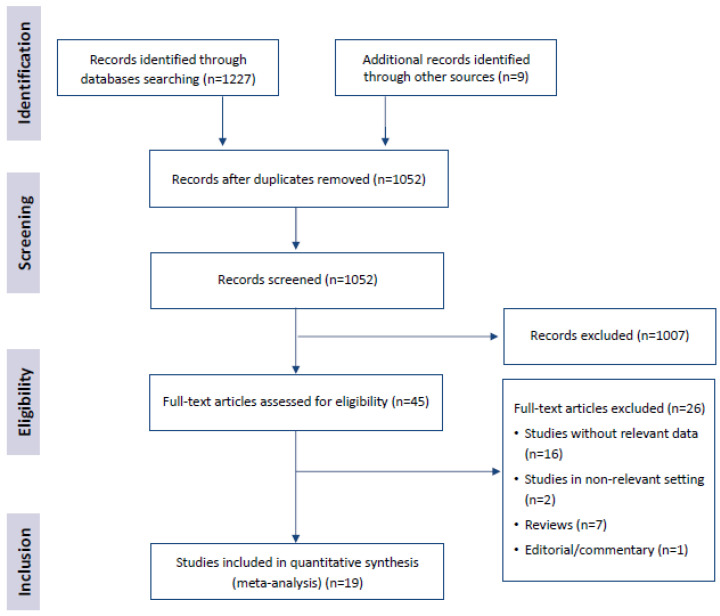
Literature search flow.

After completing the screening process as presented in Figure 1, 19 articles were included in the systematic review.

The articles were published from 1991 to 2019, applying a cross-sectional design (Table 1). The total sample included in this study was 13,280 (range 80–3433), comprising 8918 HCWs and 4362 patients or family members. Twelve articles examined violence perpetrated in nursing homes, three in home care, and four collected data in both settings. Fifteen articles focused on violence against older persons, one focused on people with dementia, two on vulnerable adults, and one on adults with disabilities.

All articles reported physical abuse prevalence, with neglect and sexual violence reported by most studies. Conversely, physical restraint was the less investigated type of violence. The majority of the articles (n = 10) collected the frequency of violence as reported by patients or their family members, nine used HCWs’ self-reported questionnaire, while six described violence witnessed by HCWs. Twelve studies focused on the prevalence of violence over the last year, while others collected lifetime or shorter prevalence.

All studies were conducted in high-income countries, including nine in the US, three in Israel, two in Germany and UK, and one each in the Czech Republic, Slovenia, and Croatia. Table 1 presents the characteristics of the included studies.

Concerning physical abuse, a total of 3378 episodes were reported over 37,337 possible responses (HCWs self-reported 450/6564, HCWs witnessed 2673/25,084, patient and family self-reported 255/5689). Episodes of Physical Restraint were 653 over 3025 possible responses (HCWs self-reported 248/1182, HCWs witnessed 202/656, patient and family self-reported 203/1187). A total of 8539 episodes of verbal abuse were reported over 30,540 possible responses (HCWs self-reported 698/3338, HCWs witnessed 6748/21,618, patient and family self-reported 1093/5584). Concerning psychological abuse, 5052 episodes were reported over 22,390 possible responses (HCWs self-reported 491/1928, HCWs witnessed 4218/17,798, patient and family self-reported 343/2664). Episodes of sexual abuse were 467 over 31688 possible responses (HCWs self-reported 33/1815, HCWs witnessed 324/26,703, patient and family self-reported 110/3170). A total of 7687 episodes of neglect were reported over 40890 possible responses (HCWs self-reported 4541/18,253, HCWs witnessed 1791/15,451, patient and family self-reported 1355/7186). Episodes of financial abuse were 1902 over 25,804 possible responses (HCWs self-reported 24/829, HCWs witnessed 1551/21,623, patient and family self-reported 327/3352).

The overall quality of the studies scored an average of 5.7/9. The complete extracted data and quality assessment are reported in Supplementary Material (Tables S2 and S3).

### 3.1. Prevalence of the Types of Violence in LTC

**Figure 2:**
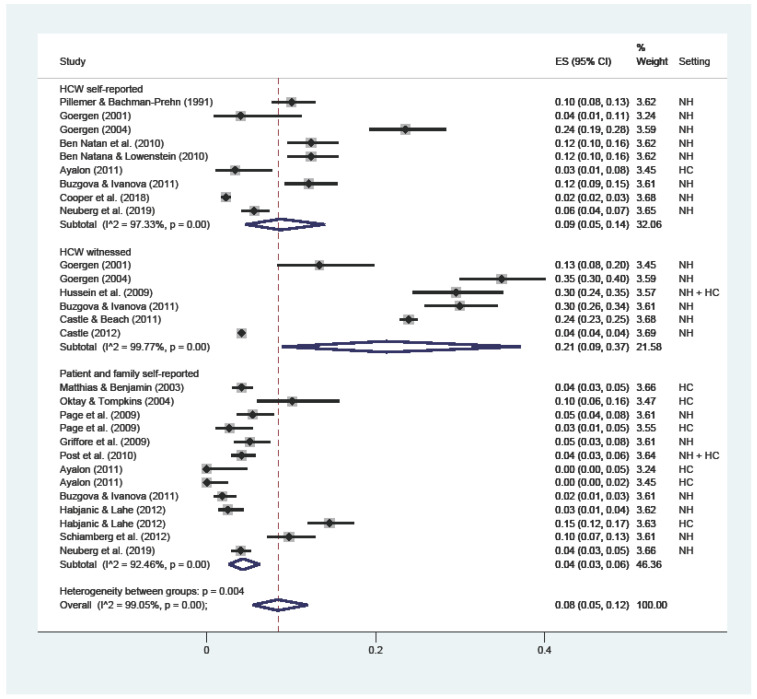
Forest plot of the prevalence of physical abuse in LTC. Subgroup meta-analyses by (Healthcare workers (HCW) self-reported, HCW witnessed, or patient and family self-reported). NH: nursing home; HC: home care.

The overall prevalence of physical abuse (Figure 2) was 8% (C.I. 5–12; I^2^ = 99%), with substantial variations among HCWs’ self-reported (9%; C.I. 5–14; I^2^ = 97%), witnessed by HCWs (21%; C.I. 9–37; I^2^ = 99%), and reported by patients or their families (4%; C.I. 3–6; I^2^ = 99%).

**Figure 3:**
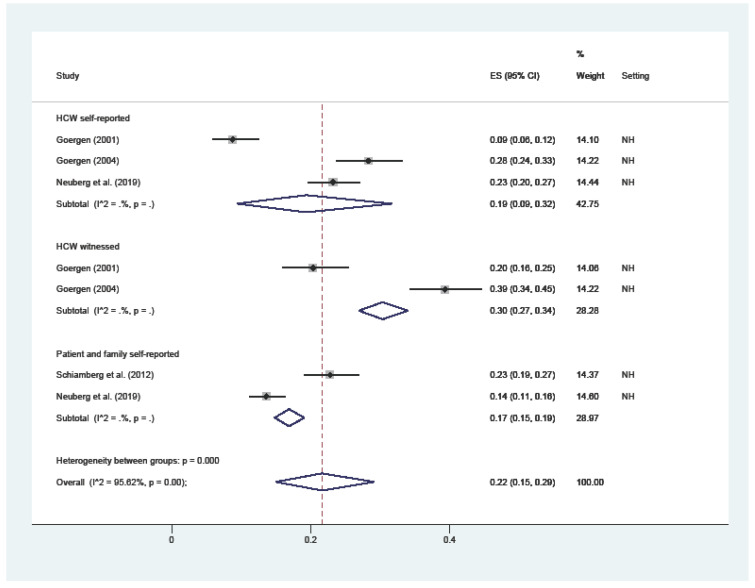
Forest plot of the prevalence of physical restraint in LTC. Subgroup meta-analyses by (Healthcare workers (HCW) self-reported, HCW witnessed, or patient and family self-reported). NH: nursing home; HC: home care.

Physical restraint (Figure 3) attained an overall 22% prevalence (C.I. 15–29; I^2^ = 96%), ranging from the 17% reported by patients or their families (C.I. 15–19; I^2^ = nc), 19% HCWs’ self-reported (C.I. 9–32; I^2^ = nc), and 30% witnessed by HCWs (C.I. 27–34; I^2^ = nc).

**Figure 4:**
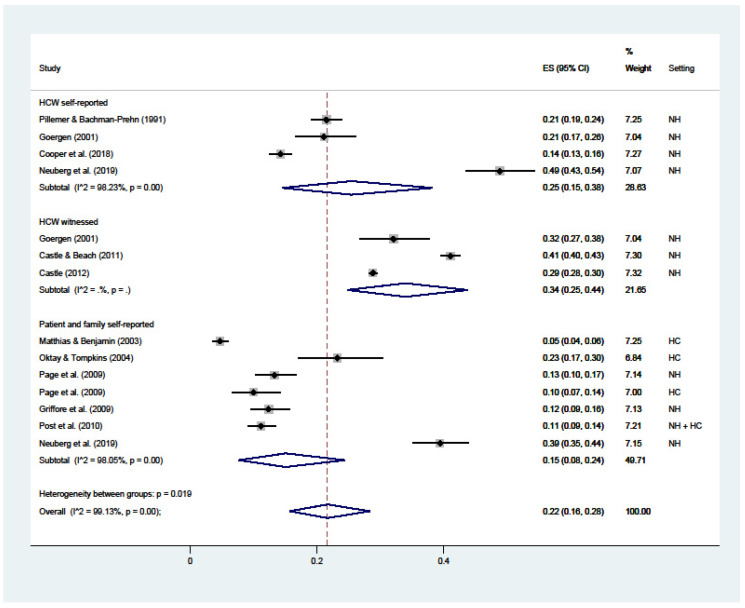
Forest plot of the prevalence of verbal abuse in LTC. Subgroup meta-analyses by (Healthcare workers (HCW) self-reported, HCW witnessed, or patient and family self-reported). NH: nursing home; HC: home care.

Verbal abuse (Figure 4) had a 22% overall prevalence (C.I. 16–28; I^2^ = 99%), with substantial variations among HCWs’ self-reported (25%; C.I. 15–38; I^2^ = 98%), witnessed by HCWs (34%; C.I. 25–44; I^2^ = nc), and reported by patients or their families (15%; C.I. 8–28; I^2^ = 99%).

**Figure 5:**
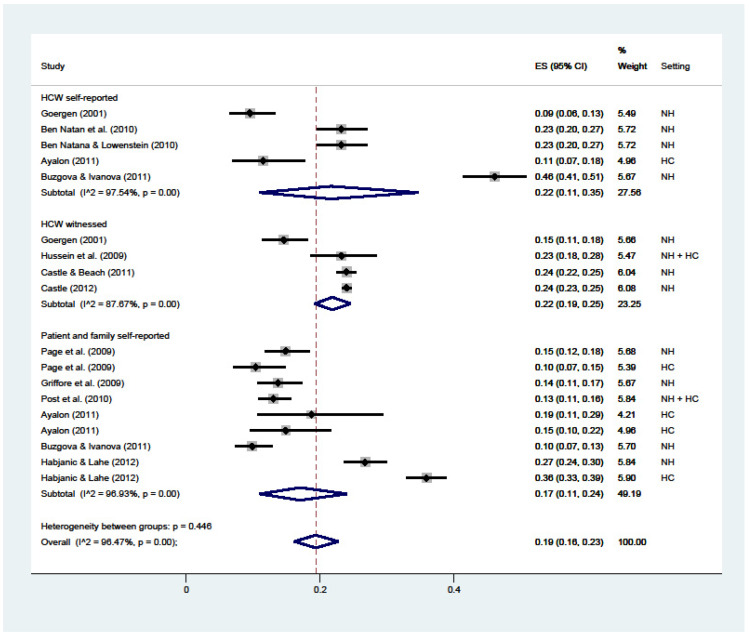
Forest plot of the prevalence of psychological abuse in LTC. Subgroup meta-analyses by (Healthcare workers (HCW) self-reported, HCW witnessed, or patient and family self-reported). NH: nursing home; HC: home care.

The overall prevalence of psychological abuse (Figure 5) was 19% (C.I. 16–23; I^2^ = 96%), attaining 22% from both HCWs’ self-reported (C.I. 11–35; I^2^ = 98%) and witnessed (C.I. 19–25; I^2^ = 88%), and 17% reported by patients or their families (C.I. 11–24; I^2^ = 96%).

**Figure 6:**
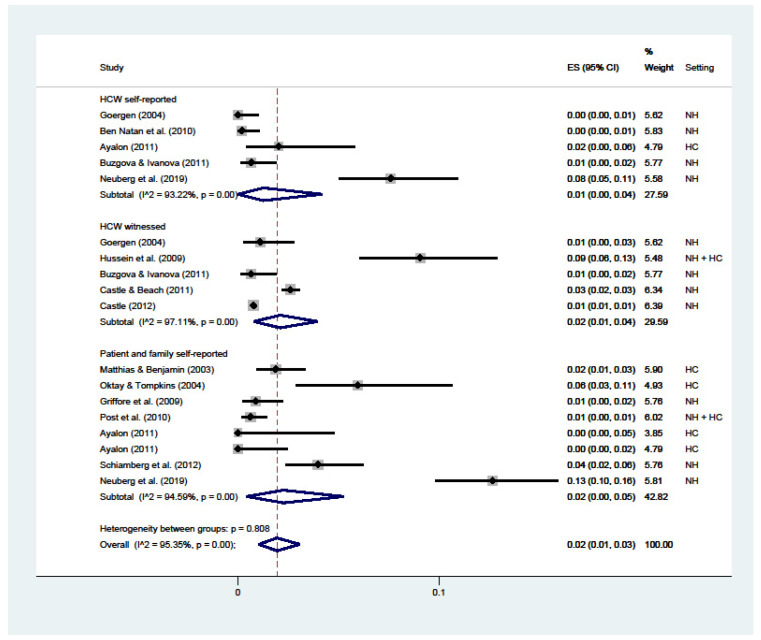
Forest plot of the prevalence of sexual abuse in LTC. Subgroup meta-analyses by (Healthcare workers (HCW) self-reported, HCW witnessed, or patient and family self-reported). NH: nursing home; HC: home care.

Sexual violence (Figure 6) had a 2% overall prevalence (C.I. 1–3; I^2^ = 95%), with a 1% prevalence from HCWs’ self-reported (C.I. 0–4; I^2^ = 93%), a 2% witnessed by HCWs (C.I. 1–4; I^2^ = 97%), and a 2% reported by patients or their families (C.I. 0–5; I^2^ = 95%).

**Figure 7:**
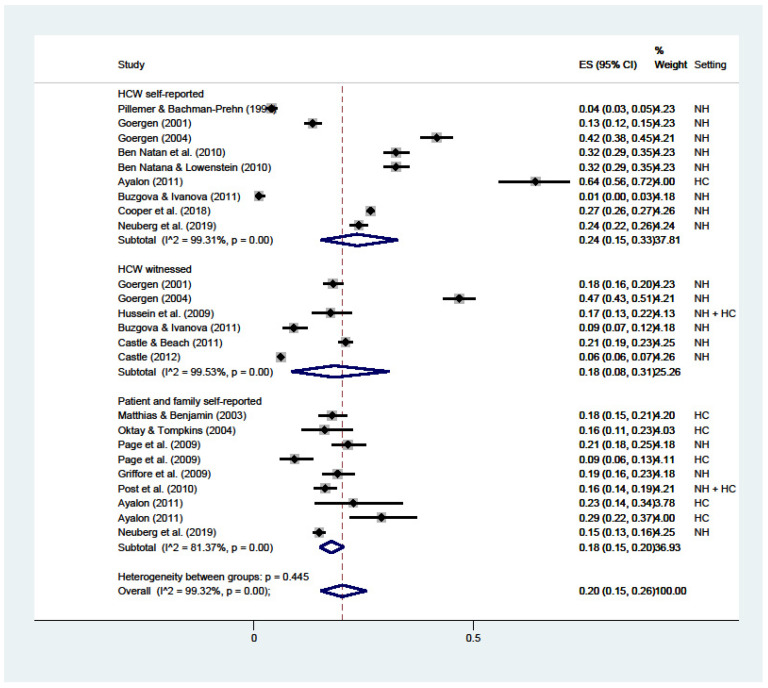
Forest plot of the prevalence of neglect in LTC. Subgroup meta-analyses by (Healthcare workers (HCW) self-reported, HCW witnessed, or patient and family self-reported). NH: nursing home; HC: home care.

Neglect (Figure 7) attained an overall 20% prevalence (C.I. 15–26; I^2^ = 99%), higher than HCWs’ self-reported (24%; C.I. 15–33; I^2^ = 99%), and at 18% for both witnessed by HCWs (C.I. 8–31; I^2^ = 99%) and reported by patients or their families (C.I. 15–20; I^2^ = 81%).

**Figure 8:**
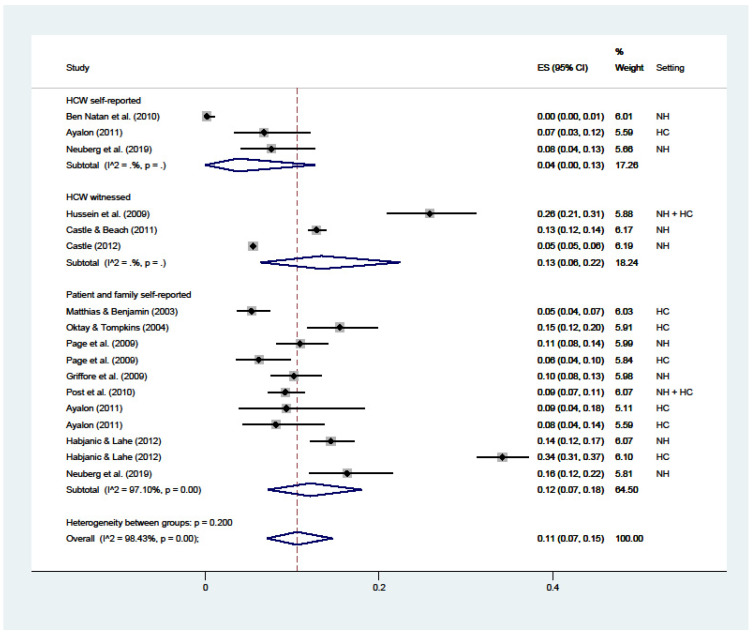
Forest plot of the prevalence of financial abuse in LTC. Subgroup meta-analyses by (Healthcare workers (HCW) self-reported, HCW witnessed, or patient and family self-reported). NH: nursing home; HC: home care.

The overall prevalence of financial abuse (Figure 8) was 11% (C.I. 7–15; I^2^ = 98%), with substantial variations among HCWs’ self-reported (4%; C.I. 0–13; I^2^ = nc), witnessed by HCWs (13%; C.I. 6–22; I^2^ = nc), and reported by patients or their families (12%; C.I. 7–18; I^2^ = 97%).

### 3.2. Subgroup Meta-Analysis of the Prevalence of Violence in Different LTC Settings

The prevalence of physical and psychological HCWs’ self-reported abuses was higher in nursing homes than in home care, while neglect (64% vs. 19%), sexual (2% vs. 1%) and financial (7% vs. 1%) abuse were higher in home care settings than nursing homes (Table 2).

As no studies assessed the prevalence of HCWs witnessing violence in home care, this data was available only for nursing homes (Table 3).

The prevalence of violence reported by patients or their families showed substantial difference among LTC settings only for verbal (21% vs. 11%) and sexual abuse (5% vs. 1%), more frequently reported in nursing homes than in home care (Table 4).

### 3.3. Subgroup Meta-Analysis of the Prevalence of Violence Stratified by Study Quality

As all meta-analyses had high overall heterogeneity (I^2^ > 80%; Cochran Q, *p* < 0.001), meta-analyses stratified by study quality were conducted. No differences in prevalence within long-term settings were found for financial abuse when analyses were stratified by study quality. The prevalence of physical abuse in nursing homes was higher in low-quality studies (13%; C.I. 5–24), compared to fair (8%; C.I. 2–16) or high-quality studies (10%; C.I. 3–19), while no differences were found in home care. Vice versa, differences in the prevalence of physical restraint were found exclusively in home care between fair (39%; C.I. 30–48) and low-quality (19%; C.I. 11–28) or high-quality studies (17%; C.I. 16–19). Verbal violence was reported more frequently in nursing homes by studies with fair (39%; C.I. 30–48) quality, whereas it was considerably lower in low-quality (17%; C.I. 16–19) and high-quality studies (19%; C.I. 11–28). The same trend was appreciable for this type of violence in home care also, despite there being only one fair-quality study (5%; C.I. 4–6) compared to two low-quality studies (15%; C.I. 11–18). Conversely, the prevalence of psychological abuse in nursing homes was higher in high-quality (25%; C.I. 8–48) or fair-quality (24%; C.I. 23–25) studies, and minor in low-quality (13%; C.I. 11–16) studies. Similarly, psychological abuse in home care had the prevalence doubled in the four fair-quality studies (20%; C.I. 8–35), compared to the only one low-quality study (10%; C.I. 7–15). Regarding the prevalence of sexual abuse, differences in nursing homes were found between fair quality studies (5%; C.I. 2–8) and low-quality (1%; C.I. 0–2) or high-quality studies (0%; C.I. 0–1). In home care, differences in prevalence of sexual abuse were found between low-quality studies (6%; C.I. 3–11) that reported a higher frequency than those with fair quality (1%; C.I. 0–2). Neglect was reported more frequently in nursing homes by studies with low (26%; C.I. 15–38) or fair (19%; C.I. 9–30) quality, whereas it was lower (12%; C.I. 3–26) in high-quality studies. An opposite trend was reported for this type of violence in home care, with a lower prevalence in low-quality (12%; C.I. 9–15) studies compared with fair-quality (33%; C.I. 14–55) studies.

Due to lack of data reporting individual and working characteristics related to HCWs perpetrating violence, no meta-regression could be performed.

### 3.4. Sensitivity Analyses

The omission of any study did not influence the pooled estimates for all types of violence. In particular, physical abuse varied from 8% to 11%; physical restraint from 17% to 23%; verbal abuse from 22% to 28%; psychological abuse from 19% to 22%; sexual abuse from 1% to 2%; neglect from 16% to 22%; and financial abuse from 8% to 11%. No statistical differences were observed.

The funnel plots showed no obvious outliers or evidence of publication bias. A small-study effect was found for all the types of violence (*p* < 0.05) except physical restraint (*p* = 0.276).

## 4. Discussion

The findings of this systematic review and meta-analysis highlighted that violence perpetrated by HCWs toward patients represents a significant concern in LTC, thereby emphasizing the need for considerable efforts to prevent and detect this phenomenon. The prevalence of violence could seem variable according to different reporting sources, requiring a comprehensive approach toward its investigation. Violence, especially that against older persons, has been identified as a significant and pervasive problem [16] both in institutional and domestic settings, accounting for more than 2500 deaths each year in Europe [5]. As promoted by WHO, elder abuse prevention should involve various actors (e.g., patients and family members) and consider dimensions such as awareness of abuse, knowledge, and behaviors [17].

The prevalence of physical abuse found in our study is comparable to the HCWs’ self-reported values identified by Yon et al., in 2017 [7], whereas it is substantially lower than values reported in the community setting [8]. Interestingly, the HCWs’ physical abuse witnessed value is higher than the results obtained from other reviews focused on older persons, but very close to the prevalence found when looking at individuals with disabilities. Our findings showed that except for financial abuse, HCWs reported having perpetrated less violence than those witnessed from their colleagues. This tendency could mean that abuse may still be considered as not serious by HCWs [16] as it is related to several drivers such as burden, job stress, burnout, staffing shortage, inadequate training, and supervision [18,19,20]. On the other hand, HCWs have continuous contact with the patients and their families [21], and might—subject to social desirability—tend to not report the violence perpetrated.

Psychological abuse estimated prevalence was high, but considerably lower than the values reported in solely institutional settings [8] and similar to those reported in the community [6,7]. Conversely, verbal abuse attained the highest prevalence among different types of violence occurring in LTC services, and not studied in previous reviews. A high prevalence of verbal and psychological abuse has been already reported by Hsieh et al., who found that most older persons experienced these types of violence when institutionalized [20]. These types of violence might be more accepted and tolerated by HCWs, and not considered as abusive. Furthermore, although psychological and verbal abuse represent a severe burden for the victim, they have limited evidence, thereby making it more challenging to report or witness. On the other hand, HCWs might feel free to report the real extent of these abuses as they may be less concerned about being punished for this behavior.

Neglect, psychological, and financial abuse are the most common types of maltreatment among older persons [22,23]. This was highlighted by our results, which showed a considerably higher prevalence of neglect than previous reviews [6,7,8]. Although no substantial differences were found in self-reported, witnessed, and patient or family self-reported prevalence of neglect, HCWs reported a higher frequency of this type of violence, especially in home care. Patients subjected to neglect experience a lower quality of life, low self-esteem, and depression [24], leading to a higher risk of mortality and hospitalization with adverse effects on both families and society [16,25]. For this reason, it is possible that HCWs may have felt guilty about not providing their perceived high-standard care, for instance, when they had limited time to complete their tasks, as in the case of home care, reporting a higher prevalence of neglect among the studies.

Although our findings assessed a higher prevalence when compared to previous reviews [7,8,9,10], they confirm that sexual abuse is the less recognized and reported form of maltreatment in LTC [26]. When involving vulnerable adults, this type of violence becomes a devastating problem with significant physical and psychological consequences for victims and their families [27,28]. Sexual abuse could be a problem for HCWs themselves [11] as many cognitively impaired patients may not only experience but also perpetrate this form of violence [26]. Interestingly, patients or their family members reported a sexual abuse prevalence that is five times higher than that reported or witnessed by HCWs. On the one hand, HCWs might not perceive their behavior as sexually abusive as some care practices performed on patients’ bodies (such as washing or toileting) are part of their working routine. On the other hand, patients might perceive these practices as abusive and report them more frequently. Nevertheless, many of them—especially older persons—have little awareness of this problem [29,30]. This could partly explain the lower prevalence of sexual abuse cases in home care, although in this setting, patients might be less likely to complain because of fear or lack of control by their families.

The prevalence of financial abuse was similar to the 14% identified by Yon et al. [8], and showed a higher frequency of reporting by patients or their relatives. This type of violence could severely impact older persons surviving on limited resources [16]. The high prevalence of financial abuse reported by patients and families could be explained by the characteristics of the setting in which they are cared for. At home or in nursing homes, belongings such as jewels, precious items, and money could be stolen while patients are in bed or absent from their room. In this case, families are aware of their relatives’ belongings and can detect if something has been stolen in a timely manner. Moreover, financial abuse could be perpetrated in nursing homes through banking activities that might be traced, making this type of violence more identifiable by families.

It is possible that some types of violence, such as verbal and psychological abuse, are being legitimized and more frequent. This could be influenced by the specific characteristics of the abuse and the possibility of detecting it. Since certain abuses are difficult to identify and have limited possibilities of legal actions against HCWs, it cannot be discounted that their prevalence has been underestimated. This could lead to a broader spread of these phenomena, with the risk that HCWs might consider them not worth reporting. In particular, the tendency to under-report some cases deemed to be scarcely relevant could lead to a considerable underestimation of the real extent of violence and its lack of monitoring in long-term care.

Patients and their relatives reported fewer cases of violence than HCWs. This tendency could be connected to a potential fear of reporting. Moreover, considering that abuse is particularly high among physically or cognitively vulnerable older persons [22,31], it could also be possible that patients are likely to have cognitive deficits affecting their ability to fully understand the abuse they have experienced [16]; they may not be aware of the abusive conduct they are subjected to. Similarly, families possibly report fewer episodes of abuse as they may be less aware of, or do not detect, evidence of such abuse. This could be because they spend less time with their relatives, if institutionalized, or patients do not report these episodes during visits. It has been found that patients who do not receive regular visits are less likely to be monitored for care quality [18,20], with poor social support, isolation, loneliness, and lack of social networks among older persons further exacerbating the abuse [32,33]. Furthermore, it might be difficult for relatives to detect signs of verbal or psychological abuse as this kind of violence does not leave visible evidence on the patient’s body. As violence leads to adverse effects on the quality of life, well-being, and mortality, especially in older adults [22,34,35,36], victims and their families should be supported in the process of reporting the abuse experienced.

Regarding the difference in violence perpetrated among long-term settings, more abuse seemed to occur within nursing homes. It may be more common for people to be vulnerable in these contexts, resulting in a greater risk of being abused since they cannot report or respond to such conduct. Furthermore, family members may not be present for much of the time, allowing HCWs to act more abusively. Finally, nursing home staff may be more stressed than HCWs working in home care, leading to tension, work overload, and conflicts related to their role that may result in inappropriate behaviors [16]. In fact, it has been found that perpetrators’ distress is a factor predisposing to a higher risk of maltreatment [16,37,38]. Compared with home care setting, lack of supervision by families could explain the higher prevalence of HCWs’ self-reported physical abuse found in nursing homes. On the other hand, in home care, families may be more often under a heavy burden of caring for their relatives, resulting in less awareness in detecting evidence of abuse, and being less sensitive to this issue. In such a setting, there were no available data on witnessed violence, presumably due to the presence of only one HCW in home care. In this regard, if we consider the difference found in nursing homes in favor of witnessed versus self-reported abuse, we should assume that potential abuse prevalence could be much higher in home care.

Conducting this systematic review and meta-analysis led to recognizing that violence towards patients has not yet been extensively studied in long-term care. Ayalon et al. came to the same conclusion, calling for more rigorous and qualitative research on maltreatment as some areas of this phenomenon still lack evidence [22]. The paucity of studies is remarkable, especially in the home care setting. Violence perpetrated in home care might be hard to study because it is influenced by many factors that are difficult to collect or are underestimated [22]. It follows that the most vulnerable population receiving home care services or unpaid care from families has so far been given little research attention, leaving violence to continue behind closed doors [39]. In this regard, the integration of routine screening for abuse in long-term care should be considered, especially for higher-risk populations [25]. Most of the articles included in this study are from northern Europe, Israel, and North America. Data from southern European countries and other continents have not yet been published, calling for more efforts to identify violence in areas where the phenomenon is poorly studied. Longitudinal studies will be needed in the future to better define the incidence, risk, protective factors, and consequences of violence in different populations [25]. Moreover, we found a trend for higher quality articles to report a lower prevalence of abuse. Thus, differences found could be referred not only to the data collection method but also to the methodological rigor applied to the research. As we found that older articles have lower quality, more recent studies could be better designed to identify episodes of violence more accurately.

More emphasis is also needed on prevention of violence by providing patients and caregivers with early detection and reporting systems. Although several reviews found that some interventions were successful in improving knowledge and attitudes about violence, these concluded that there was insufficient evidence on their effectiveness in reducing the extent of abuse [16,40,41,42]. Nevertheless, the use of multiple approaches such as education and support services seemed to be effective, as a mixed approach showed significant effects [43]. Thus, interventions using mixed approaches may be more effective, especially if combined with the use of technology (e.g., wearables, monitoring systems) that could track episodes of violence while maintaining the privacy of patients and HCWs [44]. Globally, individuals are increasingly vulnerable and at risk of violence [29], with significant economic and social consequences. Since long-term care represents one of the areas critical for public health in the coming decades, it is necessary to produce high-quality evidence on this phenomenon to guide healthcare policy choices and ensure the safety of patients and their families.

### Strengths and Limitations

This systematic review and meta-analysis present some limitations. The review of five databases and the restriction to articles published in English and Italian could have excluded some relevant studies. Moreover, the inclusion of studies conducted in high-income countries may have limited the generalizability of our findings to other medium and low-income countries. It was also not possible to stratify the results according to private or publicly funded facilities, limiting the understanding of any difference occurring between varied sources of funding. However, the adoption of a systematic approach, the presence of two independent reviewers, and the involvement of an experienced librarian in the search, screening, and extraction phases have contributed to reducing bias.

The inconsistency in the definitions and methods of measuring violence and populations (the majority of articles were focused older persons) represented an additional limitation of this review, which was contained by the use of consensus orientation regarding the adoption of operational definitions for different types of violence. This, combined with the use of self-reported data, may have contributed to an underestimation of the phenomenon and high heterogeneity in the meta-analyses. To reduce this effect, we applied a double arcsine transformation targeted at stabilizing the prevalence variance [14]. In addition, all meta-analyses performed used random-effects models. In this case, using a quality effect estimator could have maintained a lower observed variance while maintaining the correct confidence interval probability, regardless of the level of heterogeneity. This limitation was addressed by stratifying the studies by quality level, which allowed for the assessment of differences that occurred in prevalence rates obtained according to quality appraisal scores. Despite the above limitations, this systematic focused specifically on violence experienced by long-term care patients, stratifying results for setting and data source by using robust meta-analysis methods.

Lastly, the articles included in the meta-analysis were mostly dated, ranging from 1991 to 2019. This possibly influenced the results as older articles could have had a reporting bias as they attained a lower quality, while more recent articles could have been less affected by such bias. Notwithstanding, performing subgroup analyses stratified by quality reduced the effects of this limitation.

## 5. Conclusions

This systematic review and meta-analysis assessed the prevalence of violence perpetrated by HCWs towards patients in long-term care, showing differences in the type of violence, data source, and care setting. Physical restraint, verbal abuse, and neglect showed a higher prevalence, while sexual abuse and physical violence were less reported. In general, witnessed abuse is considerably higher, and violence can be underestimated, especially when investigated using self-reported tools. Except for financial abuse, patients and their families are less aware of the violence experienced. In home care, a lower prevalence of violence was generally found due to the scarcity of available studies. Our results highlight that violence in long-term care is a common problem that must be regularly screened, as it may impact patients’ health and their families’ well-being, with adverse consequences on public and social health. Further high-quality studies should be conducted, especially in home care, to assess the real extent of violence experienced by patients. Moreover, in the future, systematic reviews of qualitative studies should be conducted to gain a clearer picture of possible reasons for the occurrence of violence perpetrated by HCWs in LTC. Awareness-raising interventions and provision of penalties for types of violence that are difficult to detect are needed to contrast this phenomenon in LTC and encourage victims to report abuse.

## Figures and Tables

**Table 1:** Included studies’ characteristics.

Author(s), Year	Country	Population	Setting	Sample	Type of Violence							Data Source	Quality Assessment
					Physical Abuse	Physical Restraint	Verbal Abuse	Psychological Abuse	Sexual Abuse	Neglect	Financial Abuse		
Ayalon 2011	Israel	Older persons	Home care	371	✔			✔	✔	✔	✔	HCWs S-R; Patient or family S-R	6/9 (fair)
Ben Natan et al., 2010	Israel	Older persons	Nursing home	510	✔			✔	✔	✔	✔	HCWs S-R	8/9 (good)
Ben Natan & Lowenstein 2010	Israel	Older persons	Nursing home	510	✔			✔		✔		HCWs S-R	7/9 (fair)
Buzgova & Ivanova 2011	Czech Republic	Older persons	Nursing home	942	✔			✔	✔	✔		HCWs S-R; Witnessed; Patient or family S-R	8/9 (good)
Castle 2012	USA	Older persons	Nursing home	3433	✔		✔	✔	✔	✔	✔	Witnessed	6/9 (fair)
Castle & Beach 2011	USA	Older persons	Nursing home	832	✔		✔	✔	✔	✔	✔	Witnessed	6/9 (fair)
Cooper et al., 2018	UK	Dementia	Nursing home	1544	✔		✔			✔		HCWs S-R	8/9 (good)
Goergen 2001	Germany	Older persons	Nursing home	80	✔	✔	✔	✔		✔		HCWs S-R; Witnessed	4/9 (poor)
Goergen 2004	Germany	Older persons	Nursing home	361	✔	✔			✔	✔		HCWs S-R; Witnessed	4/9 (poor)
Griffore et al., 2009	USA	Older persons	Nursing home	452	✔		✔	✔	✔	✔	✔	Patient or family S-R	4/9 (poor)
Habjanic & Lahe 2012	Slovenia	Older persons	Nursing home and home care	300	✔			✔			✔	Patient or family S-R	5/9 (fair)
Hussein et al., 2009	UK	Vulnerable adults	Nursing home and home care	298	✔			✔	✔	✔	✔	Witnessed	3/9 (poor)
Matthias & Benjamin 2003	USA	Vulnerable adults	Home care	584	✔		✔		✔	✔	✔	Patient or family S-R	6/9 (fair)
Neuberg et al., 2019	Croatia	Older persons	Nursing home	416	✔	✔	✔		✔	✔	✔	HCWs S-R; Patient or family S-R	5/9 (fair)
Oktay & Tompkins 2004	USA	Adults with disabilities	Home care	84	✔		✔		✔	✔	✔	Patient or family S-R	4/9 (poor)
Page et al., 2009	USA	Older persons	Nursing home and home care	718	✔		✔	✔		✔	✔	Patient or family S-R	4/9 (poor)
Pillemer & Bachman-Prehn 1991	USA	Older persons	Nursing home	577	✔		✔			✔		HCWs S-R	8/9 (good)
Post et al., 2010	USA	Older persons	Nursing home and home care	816	✔		✔	✔	✔	✔	✔	Patient or family S-R	6/9 (fair)
Schiamberg et al., 2012	USA	Older persons	Nursing home	452	✔	✔			✔			Patient or family S-R	6/9 (fair)
S-R: self-reported													

**Table 2:** HCWs’ self-reported violence in different LTC settings.

	Physical Abuse	Physical Restraint	Verbal Abuse	Psychological Abuse	Sexual Abuse	Neglect	Financial
**Nursing home**	0.09 (0.05–0.15)	0.19 (0.09–0.32)	0.25 (0.15–0.38)	0.24 (0.12–0.39)	0.01 (0.00–0.05)	0.19 (0.12–0.28)	0.01 (0.00–0.02)
**Home care**	0.03 (0.01–0.09)	/	/	0.11 (0.07–0.35)	0.02 (0.00–0.06)	0.64 (0.56–0.72)	0.07 (0.03–0.12)

**Table 3:** HCWs witnessed violence in different LTC settings.

	Physical Abuse	Physical Restraint	Verbal Abuse	Psychological Abuse	Sexual Abuse	Neglect	Financial
**Nursing home**	0.20 (0.07–0.37)	0.30 (0.27–0.34)	0.34 (0.25–0.44)	0.21 (0.18–0.25)	0.01 (0.00–0.03)	0.18 (0.08–0.33)	0.07 (0.06–0.07)
**Home care**	/	/	/	/	/	/	/

**Table 4:** Patient or family self-reported violence in different LTC settings.

	Physical Abuse	Physical Restraint	Verbal Abuse	Psychological Abuse	Sexual Abuse	Neglect	Financial
**Nursing home**	0.04 (0.03–0.07)	0.17 (0.15–0.19)	0.21 (0.07–0.39)	0.16 (0.09–0.24)	0.05 (0.00–0.13)	0.18 (0.14–0.23)	0.13 (0.10–0.16)
**Home care**	0.04 (0.01–0.09)	/	0.11 (0.03–0.23)	0.19 (0.07–0.31)	0.01 (0.00–0.04)	0.18 (0.12–0.25)	0.12 (0.03–0.25)

## Data Availability

The data presented in this study are available in Table 1 and Supplementary Material—Table S2.

## Supplementary Materials

The following are available online at https://www.mdpi.com/article/10.3390/ijerph19042357/s1, Table S1: PubMed search strategy; Table S2: Included article quality assessment; Table S3: Included article quality assessment.

## References

[1] McDonald L., Beaulieu M., Harbison J., Hirst S., Lowenstein A., Bsn E.P., Wahl J. (2012). Institutional Abuse of Older Adults: What We Know, What We Need to Know. J. Elder Abus. Negl..

[2] Schiamberg L.B., Barboza G.G., Oehmke J., Zhang Z., Griffore R.J., Weatherill R.P., Von Heydrich L., Post L.A. (2011). Elder Abuse in Nursing Homes: An Ecological Perspective. J. Elder Abus. Negl..

[3] Merkur S., McDaid D., Maresso A. (2011). Meeting the Challenge of Ageing and Long-Term Care. Eurohealth.

[4] Niles N.J. (2019). Basics of the U.S. Health Care System.

[5] Sethi D., Wood S., Mitis F., Bellis M., Penhale B., Iborra Marmolejo I., Lowenstein A., Manthorpe G., Karki F.U. (2011). European Report on Preventing Elder Matreatment.

[6] Pillemer K., Burnes D., Riffin C., Lachs M.S. (2016). Elder Abuse: Global Situation, Risk Factors, and Prevention Strategies. Gerontologist.

[7] Yon Y., Mikton C.R., Gassoumis Z.D., Wilber K.H. (2017). Elder abuse prevalence in community settings: A systematic review and meta-analysis. Lancet Glob. Health.

[8] Yon Y., Ramiro-Gonzalez M., Mikton C.R., Huber M., Sethi D. (2019). The prevalence of elder abuse in institutional settings: A systematic review and meta-analysis. Eur. J. Public Health.

[9] Hughes K., Bellis M., Jones L., Wood S., Bates G., Eckley L., McCoy E., Mikton C., Shakespeare T., Officer A. (2012). Prevalence and risk of violence against adults with disabilities: A systematic review and meta-analysis of observational studies. Lancet.

[10] Jones L., Bellis M., Wood S., Hughes K., McCoy E., Eckley L., Bates G., Mikton C., Shakespeare T., Officer A. (2012). Prevalence and risk of violence against children with disabilities: A systematic review and meta-analysis of observational studies. Lancet.

[11] Clari M., Conti A., Scacchi A., Scattaglia M., Dimonte V., Gianino M.M. (2020). Prevalence of Workplace Sexual Violence against Healthcare Workers Providing Home Care: A Systematic Review and Meta-Analysis. Int. J. Environ. Res. Public Health.

[12] Phillips L.R., Guo G., Kim H. (2013). Elder Mistreatment in U.S. Residential Care Facilities: The Scope of the Problem. J. Elder Abus. Negl..

[13] Munn Z., Moola S., Lisy K., Riitano D., Tufanaru C. (2015). Methodological guidance for systematic reviews of observational epidemiological studies reporting prevalence and cumulative incidence data. Int. J. Evid.-Based Healthc..

[14] Barendregt J.J., Doi S., Lee Y.Y., Norman R.E., Vos T. (2013). Meta-analysis of prevalence. J. Epidemiol. Commun. Health.

[15] Egger M., Smith G.D., Schneider M., Minder C. (1997). Bias in meta-analysis detected by a simple, graphical test. BMJ.

[16] Choo W.Y., Hairi N.N., Othman S., Francis D.P., Baker P.R. (2013). Interventions for preventing abuse in the elderly. Cochrane Database Syst. Rev..

[17] World Health Organization (2002). The Toronto Declaration on the Global Prevention of Elder Abuse.

[18] Joshi S., Flaherty J.H. (2005). Elder Abuse and Neglect in Long-Term Care. Clin. Geriatr. Med..

[19] Wang J.-J. (2006). Psychological abuse and its characteristic correlates among elderly Taiwanese. Arch. Gerontol. Geriatr..

[20] Hsieh H.-F., Wang J.-J., Yen M., Liu T.-T. (2008). Educational support group in changing caregivers’ psychological elder abuse behavior toward caring for institutionalized elders. Adv. Health Sci. Educ..

[21] Loghmani L., Borhani F., Abbaszadeh A. (2014). Factors Affecting the Nurse-Patients’ Family Communication in Intensive Care Unit of Kerman: A Qualitative Study. J. Caring Sci..

[22] Ayalon L., Lev S., Green O., Nevo U. (2016). A systematic review and meta-analysis of interventions designed to prevent or stop elder maltreatment. Age Ageing.

[23] Lindert J., de Luna J., Torres-Gonzales F., Barros H., Ioannidi-Kopolou E., Melchiorre M.G., Stankunas M., Macassa G., Soares J.F.J. (2013). Abuse and neglect of older persons in seven cities in seven countries in Europe: A cross-sectional community study. Int. J. Public Health.

[24] Stodolska A., Parnicka A., Tobiasz-Adamczyk B., Grodzicki T. (2019). Exploring Elder Neglect: New Theoretical Perspectives and Diagnostic Challenges. Gerontology.

[25] Dong X.Q. (2015). Elder Abuse: Systematic Review and Implications for Practice. J. Am. Geriatr. Soc..

[26] Malmedal W., Iversen M.H., Kilvik A. (2015). Sexual Abuse of Older Nursing Home Residents: A Literature Review. Nurs. Res. Pract..

[27] Schneider D.C., Li X. (2006). Sexual Abuse of Vulnerable Adults: The Medical Director’s Response. J. Am. Med Dir. Assoc..

[28] Hanrahan N.P., Burgess A.W., Gerolamo A.M. (2005). Core Data Elements Tracking Elder Sexual Abuse. Clin. Geriatr. Med..

[29] Estebsari F., Dastoorpoor M., Mostafaei D., Khanjani N., Khalifehkandi Z.R., Foroushani A.R., Aghababaeian H., Taghdisi M.H. (2018). Design and implementation of an empowerment model to prevent elder abuse: A randomized controlled trial. Clin. Interv. Aging.

[30] O’Brien J.G., O’Neill D. (2011). Prevention of elder abuse. Lancet.

[31] Johannesen M., LoGiudice D. (2013). Elder abuse: A systematic review of risk factors in community-dwelling elders. Age Ageing.

[32] Acierno R., Hernandez M.A., Amstadter A.B., Resnick H.S., Steve K., Muzzy W., Kilpatrick D.G. (2010). Prevalence and Correlates of Emotional, Physical, Sexual, and Financial Abuse and Potential Neglect in the United States: The National Elder Mistreatment Study. Am. J. Public Health.

[33] Dong X., Beck T., Simon M.A. (2009). Loneliness and Mistreatment of Older Chinese Women: Does Social Support Matter?. J. Women Aging.

[34] Park H.-J. (2013). Living with ‘Hwa-byung’: The psycho-social impact of elder mistreatment on the health and well-being of older people. Aging Ment. Health.

[35] Dong X., Simon M.A. (2013). Association between Reported Elder Abuse and Rates of Admission to Skilled Nursing Facilities: Findings from a Longitudinal Population-Based Cohort Study. Gerontology.

[36] Schofield M.J., Powers J.R., Loxton D. (2013). Mortality and Disability Outcomes of Self-Reported Elder Abuse: A 12-Year Prospective Investigation. J. Am. Geriatr. Soc..

[37] Jackson S.L., Hafemeister T.L. (2011). Risk Factors Associated With Elder Abuse: The Importance of Differentiating by Type of Elder Maltreatment. Violence Vict..

[38] Schiamberg L.B., Gans D. (2000). Elder Abuse by Adult Children: An Applied Ecological Framework for Understanding Contextual Risk Factors and the Intergenerational Character of Quality of Life. Int. J. Aging Hum. Dev..

[39] Ayalon L. (2016). A triadic perspective on elder neglect within the home care arrangement. Ageing Soc..

[40] Alt K.L., Nguyen A.L., Meurer L.N. (2011). The Effectiveness of Educational Programs to Improve Recognition and Reporting of Elder Abuse and Neglect: A Systematic Review of the Literature. J. Elder Abus. Negl..

[41] Rn J.M.D., Rn M.L.M., Jogerst G.J. (2011). Elder Abuse Research: A Systematic Review. J. Elder Abus. Negl..

[42] Ploeg J., Fear J., Hutchison B., Macmillan H., Bolan G. (2009). A Systematic Review of Interventions for Elder Abuse. J. Elder Abus. Negl..

[43] Shen Y., Sun F., Zhang A., Wang K. (2021). The Effectiveness of Psychosocial Interventions for Elder Abuse in Community Settings: A Systematic Review and Meta-Analysis. Front. Psychol..

[44] Wirth T., Peters C., Nienhaus A., Schablon A. (2021). Interventions for Workplace Violence Prevention in Emergency Departments: A Systematic Review. Int. J. Environ. Res. Public Health.

