# Supplementary material for: Prevalence of Violence Perpetrated by Healthcare Workers in Long-Term Care: A Systematic Review and Meta-Analysis
Source: Int J Environ Res Public Health. 2022 Feb 18;19(4):2357. doi: 10.3390/ijerph19042357 (PMC8877976; doi:10.3390/ijerph19042357)
Supplement: Supplementary file 1 [file ijerph-19-02357-s001.zip › IJERPH Table S1.pdf]

**Table S1**. PubMed search strategy

| Database | Search string | Number of records |
|---|---|---|
| PubMed | ((("care home*"[Title/Abstract] OR "palliative care"[Mesh] OR "palliative care"[Title/Abstract] OR "home care*"[Title/Abstract] OR "home-care*"[Title/Abstract] OR "hospital at home"[Title/Abstract] OR "assisted living"[Title/Abstract] OR "assisted-living"[Title/Abstract] OR "home therapy"[Title/Abstract] OR "home drug therapy"[Title/Abstract] OR "home community care*"[Title/Abstract] OR "home care services"[Mesh] OR "home care service*"[Title/Abstract] OR "home nursing"[Mesh] OR "home nursing"[Title/Abstract] OR "Health Services for the Aged"[Mesh])) AND (("Physicians"[Mesh] OR "Physician*"[Title/Abstract] OR "Medical Staff"[Title/Abstract] OR "Health Personnel"[Mesh] OR "Health Personnel"[Title/Abstract] OR "Healthcare worker*"[Title/Abstract] OR "Health-care worker*"[Title/Abstract] OR "Health employee*"[Title/Abstract] OR "Healthcare employee*"[Title/Abstract] OR "nurses"[Mesh] OR "nurse*"[Title/Abstract] OR "caregivers"[Mesh] OR "caregiver*"[Title/Abstract] OR "care-giver*"[Title/Abstract] OR "case managers"[Mesh] OR "case manager*"[Title/Abstract] OR "GP"[Title/Abstract] OR "general practitioner*"[Title/Abstract] OR "auxiliary assistant"[Title/Abstract] OR "auxiliary nurse*"[Title/Abstract] OR "nurse assistant*"[Title/Abstract] OR "home care assistant*"[Title/Abstract] OR "home-care assistant*"[Title/Abstract] OR "home carer*"[Title/Abstract] OR "social care worker*"[Title/Abstract] OR "social worker*"[Title/Abstract] OR "community worker*"[Title/Abstract] OR "nursing aid*"[Title/Abstract]))) AND (("Aggression"[Mesh] OR "aggression*"[Title/Abstract] OR "Violence"[Mesh] OR "violence*"[Title/Abstract] OR "Workplace Violence"[Mesh] OR "Workplace Violence*" OR "Physical Abuse*"[Title/Abstract] OR "abuse*"[Title/Abstract] OR "Sex Offense*"[Title/Abstract] OR "Neglect"[Title/Abstract])) | 741 |

Limitations: Published from inception to 31-03-2021
