# Supplementary material for: Prevalence of Violence Perpetrated by Healthcare Workers in Long-Term Care: A Systematic Review and Meta-Analysis
Source: Int J Environ Res Public Health. 2022 Feb 18;19(4):2357. doi: 10.3390/ijerph19042357 (PMC8877976; doi:10.3390/ijerph19042357)
Supplement: Supplementary file 1 [file ijerph-19-02357-s001.zip › IJERPH Table S2.pdf]

Table S2. Included article data extraction															
**Study**	**Source**	**Physical Abuse**		**Physical Restraint**		**Verbal abuse**		**Psychological abuse**		**Sexual abuse**		**Neglect**		**Financial abuse**	
		**Cases**	**Resp**	**Cases**	**Resp**	**Cases**	**Resp**	**Cases**	**Resp**	**Cases**	**Resp**	**Cases**	**Resp**	**Cases**	**Resp**
Ayalon (2011)	HCWs S-R	5	148					17	148	3	148	95	148	10	148
Ayalon (2011)	Patients S-R	0	75					14	75	0	75	17	75	7	75
Ayalon (2011)	Family S-R	0	148					22	148	0	148	43	148	12	148
Ben Natan et al (2010)	HCWs S-R	63	510					118	510	1	510	330	1020	1	510
Ben Natan & Lowenstein (2010)	HCWs S-R	63	510					118	510			330	1020		
Buzgova & Ivanova (2011)	HCWs S-R	55	454					209	454	3	454	5	454		
Buzgova & Ivanova (2011)	HCWs Witn	136	454							3	454	41	454		
Buzgova & Ivanova (2011)	Patients S-R	9	488					48	488						
Castle (2012)	HCWs Witn	712	17165			4947	17165	3289	13732	160	20598	626	10299	941	17165
Castle & Beach (2011)	HCWs Witn	1591	6656			1707	4160	796	3328	130	4992	521	2496	533	4160
Cooper et al (2018)	HCWs S-R	70	3074			219	1539					2852	10721		
Goergen (2001)	HCWs S-R	3	75	27	308	64	303	29	306			174	1304		
Goergen (2001)	HCWs Witn	20	150	60	295	94	293	64	440			213	1182		
Goergen (2004)	HCWs S-R	85	361	102	361					0	361	301	722		
Goergen (2004)	HCWs Witn	126	361	142	361					4	361	338	722		
Griffore et al (2009)	Family S-R	23	452			56	452	62	452	4	452	86	452	46	452
Habjanic & Lahe (2012) – Nursing homes	Patients S-R					205	768					111	768		
Habjanic & Lahe (2012) – Home care	Patients S-R					370	1032					353	1032		
Hussein et al (2009)	HCWs Witn	88	298					69	298	27	298	52	298	77	298
Matthias & Benjamin (2003)	Patients S-R	48	1168			55	1168			11	584	104	584	31	584
Neuberg et al (2019)	HCWs S-R	48	855	119	513	167	342			26	342	408	1710	13	171
Neuberg et al (2019)	Patients S-R	49	1225	100	735	193	490			62	490	364	2450	40	245
Oktay & Tompkins (2004)	Patients S-R	17	168			39	168			10	168	27	168	52	336
Page et al (2009) – Nursing homes	Family S-R	25	458			61	458	68	458			98	458	50	458
Page et al (2009) – Home care	Family S-R	7	260			26	260	27	260			24	260	16	260
Pillemer & Bachman-Prehn (1991)	HCWs S-R	58	577			248	1154					46	1154		
Post et al (2010)	Family S-R	33	795			88	788	102	783	5	801	128	791	73	794
Schiamberg et al (2012)	Family S-R	44	452	103	452					18	452				

Resp: number of possible responses; HCWs: healthcare workers; S-R: self-reported
