# Supplementary material for: Prevalence of Violence Perpetrated by Healthcare Workers in Long-Term Care: A Systematic Review and Meta-Analysis
Source: Int J Environ Res Public Health. 2022 Feb 18;19(4):2357. doi: 10.3390/ijerph19042357 (PMC8877976; doi:10.3390/ijerph19042357)
Supplement: Supplementary file 1 [file ijerph-19-02357-s001.zip › IJERPH Table S3.pdf]

Table S3. Included article quality assessment

Study	Q1	Q2	Q3	Q4	Q5	Q6	Q7	Q8	Q9	Total (Max: 5)
Ayalon (2011)	Yes	Yes	No	Yes	Unclear	Yes	Yes	Yes	Unclear	6/9 (fair)
Ben Natan et al (2010)	Yes	Yes	Yes	Yes	Unclear	Yes	Yes	Yes	Yes	8/9 (good)
Ben Natan & Lowenstein (2010)	Yes	Yes	Yes	Yes	Unclear	Unclear	Yes	Yes	Yes	7/9 (fair)
Buzgova & Ivanova (2011)	Yes	Yes	Yes	Yes	Unclear	Yes	Yes	Yes	Yes	8/9 (good)
Castle (2012)	Yes	Yes	Yes	Unclear	Unclear	Yes	Yes	Yes	Unclear	6/9 (fair)
Castle & Beach (2011)	Yes	Yes	Yes	Unclear	Unclear	Yes	Yes	Yes	Unclear	6/9 (fair)
Cooper et al (2018)	Yes	Yes	Yes	Yes	Unclear	Yes	Yes	Yes	Yes	8/9 (good)
Goergen (2001)	Yes	Yes	No	Unclear	Unclear	Yes	Yes	Unclear	Unclear	4/9 (poor)
Goergen (2004)	Yes	Yes	No	Unclear	Unclear	Yes	Yes	Unclear	Unclear	4/9 (poor)
Griffore et al (2009)	Yes	Yes	Yes	Yes	Unclear	Unclear	Unclear	Unclear	Unclear	4/9 (poor)
Habjanic & Lahe (2012)	Yes	Yes	Yes	Yes	Unclear	Unclear	Unclear	Yes	Unclear	5/9 (fair)
Hussein et al (2009)	Yes	Unclear	Yes	Unclear	Unclear	Unclear	Unclear	Yes	Unclear	3/9 (poor)
Matthias & Benjamin (2003)	Yes	Yes	Yes	Yes	Unclear	Yes	Unclear	Yes	Unclear	6/9 (fair)
Neuberg et al (2019)	Yes	Unclear	Yes	Unclear	Unclear	Yes	Yes	Yes	Unclear	5/9 (fair)
Oktay & Tompkins (2004)	Yes	Yes	No	Yes	Unclear	Yes	Unclear	Unclear	Unclear	4/9 (poor)
Page et al (2009)	Yes	Unclear	Yes	Unclear	Unclear	Yes	Unclear	Yes	Unclear	4/9 (poor)
Pillemer & Bachman-Prehn (1991)	Yes	Yes	Yes	Yes	Unclear	Yes	Yes	Yes	Yes	8/9 (good)
Post et al (2010)	Yes	Yes	Yes	Yes	Unclear	Yes	Unclear	Yes	Unclear	6/9 (fair)
Schiamberg et al (2012)	Yes	Yes	Yes	Yes	Unclear	Yes	Unclear	Unclear	Unclear	5/9 (fair)
